# Fiber-optic bronchoscopy for the removal of an impacted foreign body in the left mainstem bronchus: a case report with review of literature

**DOI:** 10.1093/jscr/rjaf254

**Published:** 2025-04-28

**Authors:** Ali Al Bshabshe, Maraam Al Qout

**Affiliations:** Department of Adult Critical Care, College of Medicine, King Khalid University, Abha 61421, Saudi Arabia; General Practitioner, Ministry of Health, Abha Health Sector, Abha 62521, Saudi Arabia

**Keywords:** fiber-optic bronchoscopy, left mainstem bronchus, impacted foreign body

## Abstract

Tracheobronchial foreign body aspiration is a common worldwide condition that often evolves into life-threatening complications. While it is most frequently observed in pediatric patients, it can also occur in adults. Herein, we report a case of using fiberoptic bronchoscopy to remove an impacted foreign body from the left mainstem bronchus of a 47-year-old female intensive care unit patient, underscoring the efficacy and minimally invasive nature of this modality in managing complex airway obstruction. This paper includes a literature review of previously reported cases, providing a broader context for the management of tracheobronchial foreign body airway aspirations.

## Introduction

Tracheobronchial foreign body (FB) aspiration is a global health concern that can lead to life-threatening complications if not promptly managed [1]. While more common in children, it also affects adults, emphasizing its relevance across all ages [2]. The varied clinical presentations highlight the need for timely recognition and intervention to reduce morbidity and improve outcomes [3]. This case of a 47-year-old female with FB aspiration demonstrates the effectiveness of fiberoptic bronchoscopy for removing impacted foreign bodies in an intensive care unit (ICU) setting.

## Case presentation

This case report details the condition of a 47-year-old Saudi female ICU patient who suffered from FB airway aspiration. Her medical history was significant for diabetes mellitus and presented with diabetic ketoacidosis and loss of consciousness, which prompted her immediate admission to the ICU on 27 September 2022. The diagnosis of autoimmune encephalitis was made.

**Figure 1:**
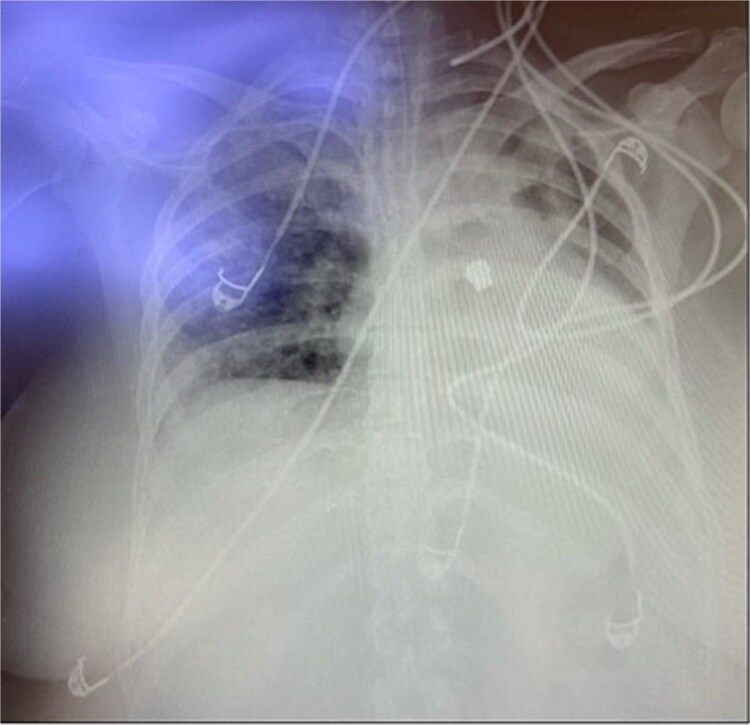
X-ray of the chest showing the foreign body in the left chest.

Throughout her 1-month hospitalization, she underwent two extubation trials, both of which were unsuccessful, necessitating reintubation. Extubation trials were unsuccessful due to weak respiratory muscles resulting from prolonged intubation and accumulation of secretions. After the second reintubation, a routine post-intubation chest X-ray incidentally revealed a FB located in the left middle zone of her left lung (Fig. 1). Despite this finding, the patient remained clinically stable with no significant changes in her oxygenation levels or ventilator parameters. The FB was suspected to be a dental bridge, likely aspirated during the second reintubation procedure.

After obtaining informed and written consent, an emergency fiberoptic bronchoscopy was performed in the ICU on 20 October 2022. During the procedure, a FB was visualized at the origin of left mainstem bronchus. The decision was to extract the FB using endobronchial basket. However, the basket could not be deployed and opened in the left main bronchus due to the small size of the bronchus. To address this issue, the team utilized an endoscopic retrograde cholangiopancreatography (ERCP) balloon to mobilize the dental bridge from the left mainstem bronchus to the carina, facilitating the removal of the FB. Following that, the endobronchial basket was passed and opened to capture the dental bridge.

During the pulling of the FB, it was during the extraction process, it was observed that the dental bridge was larger than the inner diameter of the endotracheal tube (ETT). For that reason, a new ETT and a video laryngoscope were prepared. The ETT was then removed along with the bronchoscope and the endobronchial basket as one unit. Using a Glidescope video laryngoscope, the new ETT was successfully passed through the vocal cords. The correct positioning of the ETT was confirmed by bronchoscopy, ensuring optimal oxygenation.

**Figure 2:**
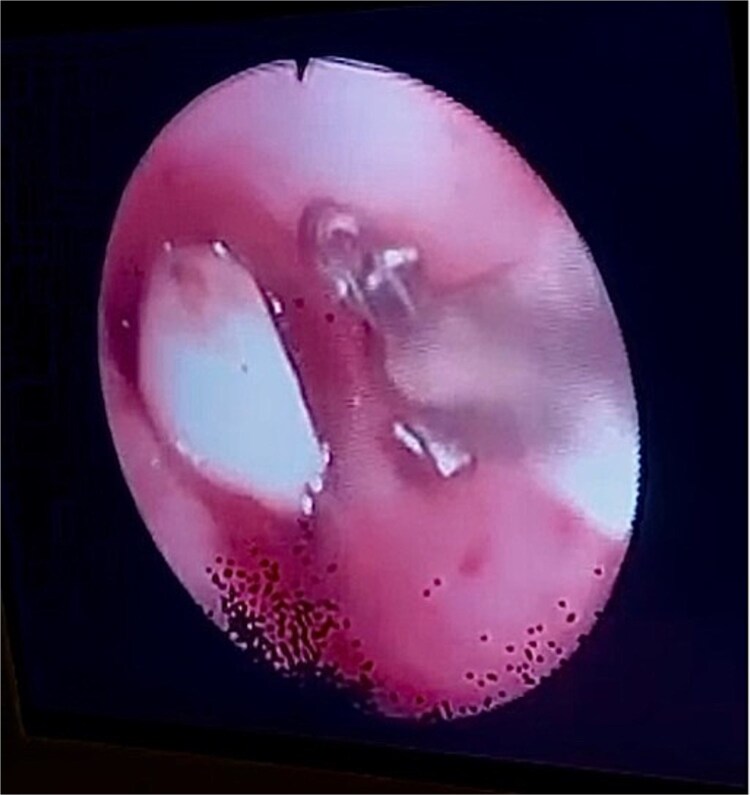
Bronchoscopy view showing the dental bridge lodged in the left mainstem bronchus.

**Figure 3:**
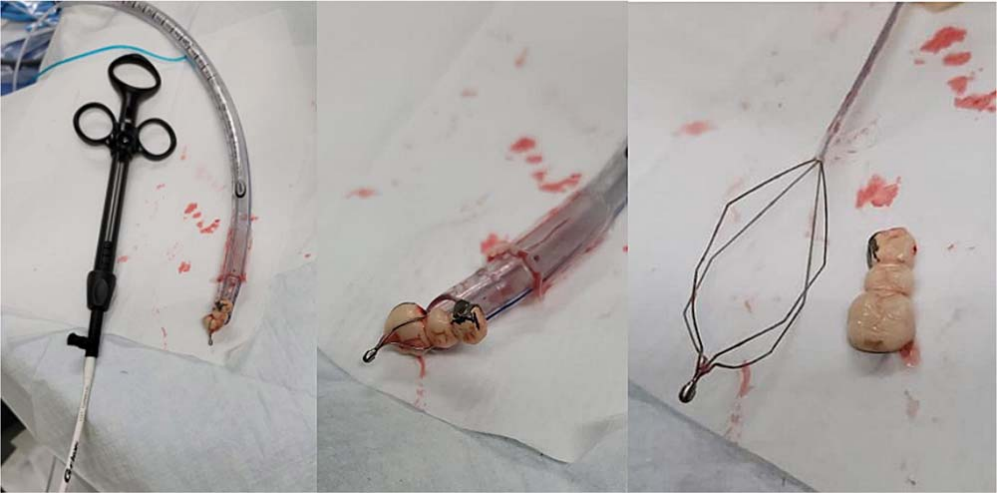
The dental bridge extracted by flexible bronchoscope.

Following the procedure, the patient underwent a percutaneous tracheostomy and was gradually weaned off the mechanical ventilator. After a 2-month stay in the ICU, the patient was discharged to a rehabilitation center for further recovery (Figs 2 and 3).

## Discussion

FB aspiration in the tracheobronchial tree is commonly observed in children, with an increasing incidence among adults [4]. Ng *et al.*, 2019, identified cough and recurrent pneumonias as the most common presentations in adults [5]. Diagnosis is typically based on history, physical examination, and radiological investigations, though FB aspiration can occasionally be clinically insignificant. This case highlights a patient who was asymptomatic due to intubation and mechanical ventilation, with the diagnosis made incidentally through a routine ICU chest X-ray. This underscores the importance of imaging in ICU patients, especially those with multiple intubation attempts. However, frequent imaging may not be necessary or cost-effective in all cases, particularly if oxygenation and ventilator parameters remain stable [6].

Management of tracheobronchial FB aspiration often involves choosing between rigid and flexible bronchoscopy, each with its strengths and limitations [7]. Since 1897, rigid bronchoscopy has been a key tool for FB extraction, though it requires experienced pulmonologists [8]. Flexible bronchoscopy, introduced in the 1970s, has demonstrated an 89% success rate in removing FBs in 300 cases [9]. It is increasingly preferred in stable patients due to ease of use, lower cost, and the ability to perform under local anesthesia, avoiding general anesthesia [10]. Its versatility, combined with tools like forceps, snares, and baskets, makes it a valuable option for airway management [11, 12].

The literature documents flexible bronchoscopy’s success across varied scenarios, FB types, and demographics (Table 1). Most cases report successful retrieval, with only two documented failures. Complications include one case of sepsis resulting in mortality after 70 days of hospitalization [15]. Previously reported cases predominantly involved male patients, often following motor vehicle accidents, highlighting variability in extraction techniques and tools used.

**Table 1:** Review of previously reported cases of airway FB aspiration removed by flexible bronchoscopy.

**Author**	En-Kwei Tang *et al.*	Wen-lin Xiao *et al.*	Mao Zhang *et al.*	Mao Zhang *et al.*	Mao Zhang *et al.*	Karan Madan *et al.*	Mohd Othman *et al.*	Dong Kim *et al.*	Dong Kim *et al.*	Ali Al Bshabshe	Ali Al Bshabshe *et al.*
**Year**	2005	2008	2011	2011	2011	2012	2013	2014	2014	2016	2022
**Age/gender**	78/Male	22/Male	23/Male	26/Male	25/Male	22/Male	46/Male	48/Male	69/Male	Young male	24/Male
**Medical history/ risk factors**	Sudden onset of central cyanosis, cons- ciousness alteration, and a seizure episode	Motor vehicle accident with multiple crush injuries	Motor vehicle accident with severe injury	Motor vehicle accident with polytrauma	Motor vehicle accident with polytrauma	Motor vehicle accident with polytrauma and maxillofacial trauma	Motor vehicle accident Intubated	Motor vehicle accident with maxillofacial trauma	Polytrauma after falling from stairs	Motor vehicle accident, ICU patient intubated on mechanical ventilator.	Polytrauma with maxillofacial injuries
**Type of foreign body**	Denture	2 teeth	Tooth	Tooth	Tooth	Tooth	Tooth	2 teeth	Multiple teeth	Broken glass	Tooth
**Site of impaction**	Right bronchial tree	Right bronchial tree	Right bronchial tree	Left lung lobe	Right bronchial tree	Left main bronchus	Right lower bronchus	Right main bronchus	Left bronchus	Right main stem bronch	Right bronchus intermedius
**Clinical presentation**	Referred to local hospital. Initially asymptomatic.	Asymptomatic	ISS = 50 AIS = 5	Cough ISS = 54 AIS = 5	ISS = 41 AIS = 4	O2 saturation not improved despite mechanical ventilation	Bilateral pneumothorax	Asymptomatic	Pneumonia	Asymptomatic	GSC = 5 HR = 113 BP = 153/80 mmHg RR = 13 bpm
**Diagnostic modality**	Chest X-ray	Chest CT	Chest CT	Chest X-ray	Chest CT	Chest X-ray	Chest X-ray	Chest X-ray	Chest X-ray	Chest CT scan	Chest X-ray
**Bronchoscopy type**	Flexible bronchoscope through a laryngeal mask airway, after failure of flexible bronchoscope alone.	Flexible bronchoscope	Both flexible and rigid failed	Both flexible and rigid failed	Flexible bronchoscope	Rigid bronchoscope after failure of flexible bronchoscope	Rigid bronchoscope after failure of flexible bronchoscope	Flexible bronchoscope	Flexible bronchoscope	Flexible bronchoscope	Flexible bronchoscope
**Removal technique/ tools**	Laryngeal mask airway and grasping forceps	Alligator jaw and cup forceps and wire basket.	None	None	None	None	Tracheostomy Optical forceps	Tracheostomy	Tracheostomy & Magill forceps	Stone retrieval basket	Rat tooth alligator jaw
**Procedure outcome**	Successful	Successful	Failed	Failed but the tooth was expectorated from tracheostomy tube due to vigorous cough	Successful	Successful	Successful	Successful	Successful	Successful	Successful
**Duration of hospital stay**	14 days	_	70 days	12 days	8 days	30 days	_	5 days	_	60 days	12 days
**Follow-up and long-term outcomes**	Discharged home	Discharged home	Died	Discharged home	Discharged home	Discharged home and remained asymptomatic	Discharged home	Discharged home	Discharged home	Discharged to rehabilitation center	Discharged home
**Complications encountered**	None	None	Sepsis	None	None	None	None	None	None	None	None
**Reference**	[13]	[14]	[15]	[15]	[15]	[16]	[17]	[18]	[18]	[19]	[20]

Abbreviations: ICU = intensive care unit, ISS = injury severity score, AIS = abbreviated injury score, GCS = Glasgow coma scale, HR = heart rate, BP = blood pressure, RR = respiratory rate, CT = computed tomography.

Rigid bronchoscopy remains the preferred choice in emergencies requiring rapid FB removal [13]. While flexible bronchoscopy succeeded in this case, challenges like deploying the endobronchial basket in a small bronchus raise questions about its suitability. Rigid bronchoscopy is often more effective for quick removal [14]. FBs are commonly found in the right bronchial tree due to its anatomy [16]. However, in this case, the FB was lodged in the left mainstem bronchus. Using a flexible bronchoscope, the team mobilized the FB to the carina with an ERCP balloon, then extracted it using an endobronchial basket. The ERCP balloon’s innovative use highlights the adaptability of endoscopic techniques. However, its broader applicability needs further validation, as it requires specialized skills and equipment [17].

The patient’s extubation failures, due to weak respiratory muscles and secretion accumulation, highlight the importance of comprehensive respiratory therapy and early mobilization to avoid prolonged intubation complications. Patients with complex medical conditions, such as diabetic ketoacidosis and autoimmune encephalitis, have an inherently higher risk of extubation failure [18]. While tooth aspiration has been linked to acute respiratory failure [19], this patient experienced no such complications. Flexible bronchoscopy facilitated successful FB removal without adverse events, reaffirming its efficacy in managing lower respiratory FBs [20].

## Conclusion

In conclusion, this report highlights the effectiveness of flexible fiber-optic bronchoscopy in managing airway foreign bodies. It stresses the importance of prompt intervention, raising awareness among healthcare providers about FB aspiration in intubated patients, and reinforces the critical role of fiber-optic bronchoscopy in ensuring safe and effective management.
